# Water in the terrestrial planet-forming zone of the PDS 70 disk

**DOI:** 10.1038/s41586-023-06317-9

**Published:** 2023-07-24

**Authors:** G. Perotti, V. Christiaens, Th. Henning, B. Tabone, L. B. F. M. Waters, I. Kamp, G. Olofsson, S. L. Grant, D. Gasman, J. Bouwman, M. Samland, R. Franceschi, E. F. van Dishoeck, K. Schwarz, M. Güdel, P.-O. Lagage, T. P. Ray, B. Vandenbussche, A. Abergel, O. Absil, A. M. Arabhavi, I. Argyriou, D. Barrado, A. Boccaletti, A. Caratti o Garatti, V. Geers, A. M. Glauser, K. Justannont, F. Lahuis, M. Mueller, C. Nehmé, E. Pantin, S. Scheithauer, C. Waelkens, R. Guadarrama, H. Jang, J. Kanwar, M. Morales-Calderón, N. Pawellek, D. Rodgers-Lee, J. Schreiber, L. Colina, T. R. Greve, G. Östlin, G. Wright

**Affiliations:** 1 Max Planck Institute for Astronomy, Heidelberg, Germany; 2 STAR Institute, Université de Liège, Liège, Belgium; 3 Université Paris-Saclay, CNRS, Institut d’Astrophysique Spatiale, Orsay, France; 4 Department of Astrophysics/IMAPP, Radboud University, Nijmegen, the Netherlands; 5 SRON Netherlands Institute for Space Research, Leiden, the Netherlands; 6 Kapteyn Astronomical Institute, Rijksuniversiteit Groningen, Groningen, the Netherlands; 7 Department of Astronomy, Stockholm University, AlbaNova University Center, Stockholm, Sweden; 8 Max-Planck Institut für Extraterrestrische Physik (MPE), Garching, Germany; 9 Institute of Astronomy, KU Leuven, Leuven, Belgium; 10 Leiden Observatory, Leiden University, Leiden, the Netherlands; 11 Dept. of Astrophysics, University of Vienna, Vienna, Austria; 12 ETH Zürich, Institute for Particle Physics and Astrophysics, Zürich, Switzerland; 13 Université Paris-Saclay, Université Paris Cité, CEA, CNRS, AIM, Gif-sur-Yvette, France; 14 Dublin Institute for Advanced Studies, Dublin, Ireland; 15 Centro de Astrobiología (CAB), CSIC-INTA, Villanueva de la Cañada, Spain; 16 LESIA, Observatoire de Paris, Université PSL, CNRS, Sorbonne Université, Université de Paris, Meudon, France; 17 INAF – Osservatorio Astronomico di Capodimonte, Napoli, Italy; 18 UK Astronomy Technology Centre, Royal Observatory Edinburgh, Edinburgh, UK; 19 Chalmers University of Technology, Onsala Space Observatory, Onsala, Sweden; 20 SRON Netherlands Institute for Space Research, Groningen, the Netherlands; 21 Space Research Institute, Austrian Academy of Sciences, Graz, Austria; 22 TU Graz, Fakultät für Mathematik, Physik und Geodäsie, Graz, Austria; 23 Centro de Astrobiología (CAB, CSIC-INTA), Carretera de Ajalvir, Torrejón de Ardoz, Spain; 24 DTU Space, Technical University of Denmark, Kgs. Lyngby, Denmark; 25 Department of Astronomy, Oskar Klein Centre, Stockholm University, Stockholm, Sweden

**Keywords:** Astrophysical disks, Astrophysical dust, Exoplanets

## Abstract

Terrestrial and sub-Neptune planets are expected to form in the inner (less than 10 au) regions of protoplanetary disks^1^. Water plays a key role in their formation^2–4^, although it is yet unclear whether water molecules are formed in situ or transported from the outer disk^5,6^. So far Spitzer Space Telescope observations have only provided water luminosity upper limits for dust-depleted inner disks^7^, similar to PDS 70, the first system with direct confirmation of protoplanet presence^8,9^. Here we report JWST observations of PDS 70, a benchmark target to search for water in a disk hosting a large (approximately 54 au) planet-carved gap separating an inner and outer disk^10,11^. Our findings show water in the inner disk of PDS 70. This implies that potential terrestrial planets forming therein have access to a water reservoir. The column densities of water vapour suggest in-situ formation via a reaction sequence involving O, H_2_ and/or OH, and survival through water self-shielding^5^. This is also supported by the presence of CO_2_ emission, another molecule sensitive to ultraviolet photodissociation. Dust shielding, and replenishment of both gas and small dust from the outer disk, may also play a role in sustaining the water reservoir^12^. Our observations also reveal a strong variability of the mid-infrared spectral energy distribution, pointing to a change of inner disk geometry.

## Abstract (web summary)

Observations with the sensitive mid-infrared spectrometer MIRI on board JWST reveal the presence of a water vapour reservoir in the terrestrial plant-forming zone of the young planetary system PDS 70.

## Main

**Fig. 1:**
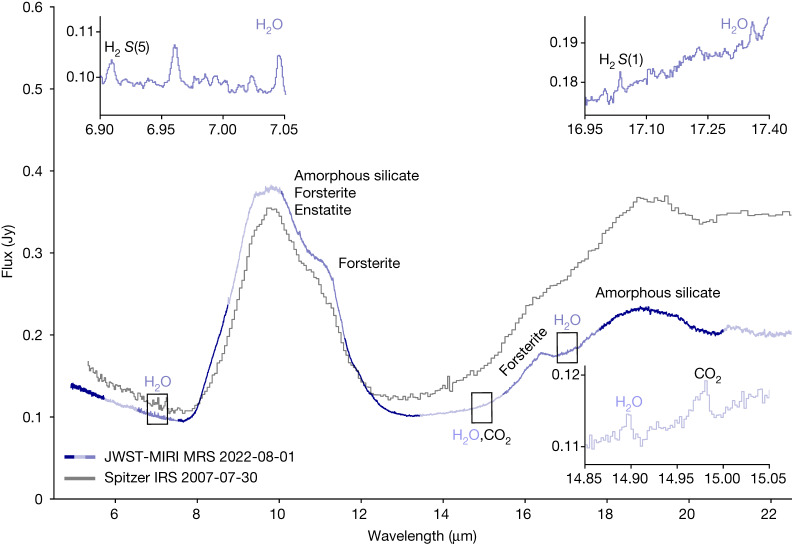
JWST-MIRI MRS spectrum of PDS 70. The spectrum is a composite of the colour-coded short, medium and long sub-bands of the four MIRI MRS Integral Field Units (IFUs)^13^. The Spitzer-IRS spectrum of PDS 70 is also shown in grey. The major dust features are labelled. The spectrum is dominated by exceptionally prominent silicate emission at 10 and 18 μm and it clearly shows a number of crystalline dust features. The much higher sensitivity and spectral resolution of MIRI MRS compared with Spitzer-IRS allows us to detect an inner disk gas reservoir by showing weak emission of water vapour and carbon dioxide as well as two molecular hydrogen lines. The insets show the ro-vibrational and rotational transitions of ortho- and para-H_2_O, the molecular hydrogen H_2_*S*(1) and *S*(5) rotational lines, and the *ν*_5_ bending mode of CO_2_.

**Extended Data Fig. 1:**
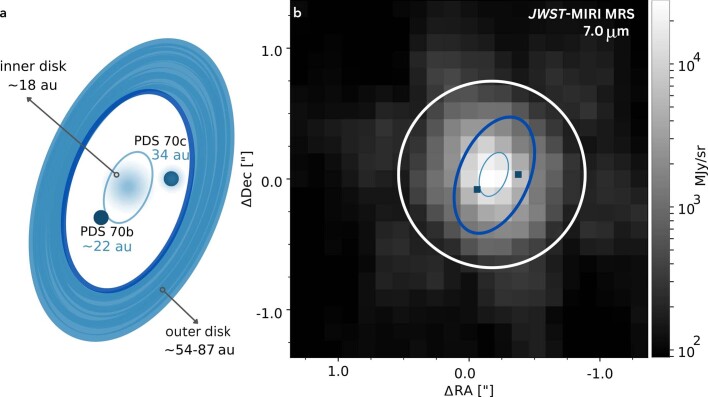
The architecture of the PDS 70 system. **a**, Schematic representation of the locations of the inner and outer disk of PDS 70 indicated as teal and blue ellipses. The protoplanets PDS 70 b and PDS 70 c are shown as blue dots. **b**, Main components of the schematic of the system on top of a MIRI-MRS IFU image at 7.0 *μ*m, illustrating the size of the system with respect to the 2.5-FWHM aperture used for spectro-photometric extraction (white circle). The latter linearly increases with wavelength.

  Observations of  PDS 70 were taken with the JWST Mid-InfraRed Instrument (MIRI)^13,14^ Medium Resolution Spectrometer^15^ (MRS; spectral resolving power *R* ≈ 1,600–3,400) as part of the guaranteed time MIRI mid-INfrared Disk Survey (MINDS; see Methods and Extended Data Fig. 1). The complete spectrum of PDS 70 shows several distinct traits (Fig. 1), which stands out with respect to other T Tauri disks^16,17^.

A significant flux offset—up to a factor of 1.5 at wavelengths beyond 18 μm—is found between the MIRI and the archival Spitzer InfraRed Spectrograph (IRS) low-resolution (*R* ≈ 60–100) spectra recorded with 15 years and one day time difference. This discrepancy is too large to be explained by calibration uncertainties; the absolute uncertainty for both IRS and MIRI is approximately 5% for the 4.9–22.5 μm range^18^. Similarly, the difference in aperture size of the two spectrographs cannot account for such an offset. Hence, with the current MIRI data reduction, time variability is the most likely explanation for the observed flux differences.

Variability in the mid-infrared observed with Spitzer-IRS has been mainly attributed to short-wavelength stellar irradiation or to dynamical changes in the inner disk geometry due to the presence of planets^19,20^. In the case of PDS 70, stellar irradiation is excluded as it would cause an overall increase or decrease in flux, contrary to what is observed with the Wide-field Infrared Survey Explorer (WISE) time-series observations (Extended Data Fig. 6). PDS 70 is known to be in a late stage of accretion—with an estimated waning accretion rate^21,22^ of approximately 10^−10^ *M*_⊙_ yr^−1^ making it unlikely to explain the significant flux difference. Changes in the scale height of the inner disk wall emitting at shorter wavelengths (approximately 2–8 μm) can be responsible for shadowing the disk material located further out, resulting in less emission at wavelengths beyond 18–20 μm (also referred to as ‘seesaw-like’ variability^19^). However, for PDS 70 a complete seesaw-like profile is not observed as there is no corresponding increase in flux at the shorter wavelengths. Time variability is also supported by the above-mentioned WISE observations, which indicate that such variability occurs on short timescales (of at most 1 yr) and that it may indeed be attributed to occulting material located close to the star (approximately 1 au).

**Fig. 2:**
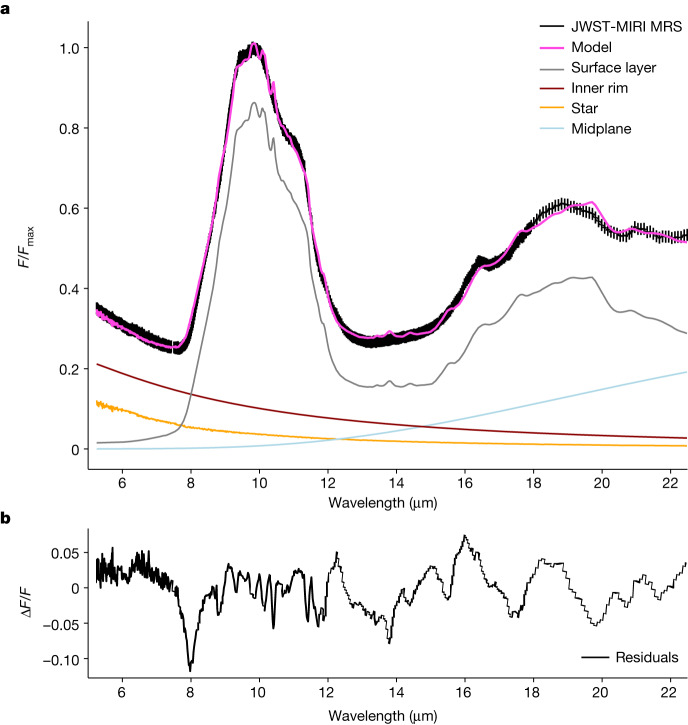
Dust continuum fit to the MIRI spectrum of PDS 70. **a**, The disk model has three spectral components: an inner rim, an optically thick midplane disk layer and an optically thin warm disk surface layer. The stellar photospheric emission is represented by a stellar atmosphere model (see Methods for further details). The surface layer component dominates the MIRI spectrum in the 8–22.5 μm wavelength range. Its temperature is constrained to be between 400 and 600 K. The silicate emission at 8–12 μm is consistent with a population of optically thin dust grains with typical sizes of 0.1–2 μm. A significant contribution from an optically thick dust component is excluded because of the high silicate peak/continuum ratio of approximately 4 (ref. ^31^). **b**, The residuals on the dust continuum fit.

The MIRI spectrum of PDS 70 clearly shows the presence of silicate dust grains that have undergone significant thermal processing (Fig. 1). We attribute the crystalline dust features to enstatite at 9.40 μm and forsterite at 11.30  and 16.40 μm. The observed dust continuum is well reproduced with a three-component disk model, with a 400–600 K surface layer accounting for the bulk of the observed emission (Fig. 2).

**Fig. 3:**
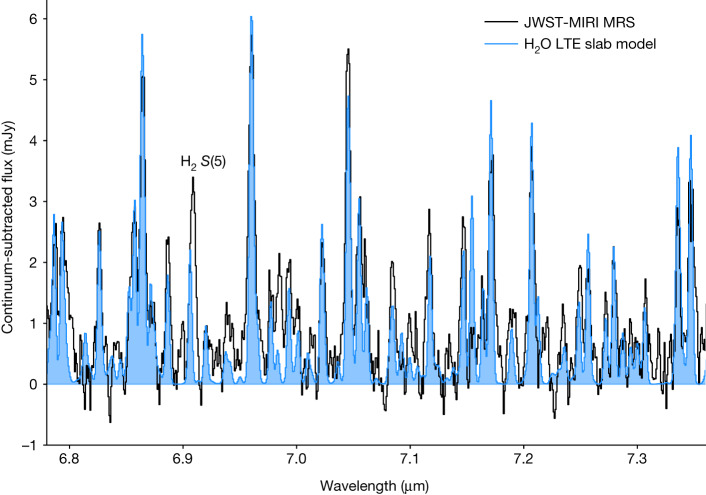
Continuum-subtracted spectrum showing H_2_O emission in the 7 μm region and the best-fit LTE slab model. The best-fit model (blue) has *T* = 600 K, *N*(H_2_O)= 1.4 × 10^18 ^cm^−2^ and *R* = 0.047 au. The molecular hydrogen H_2_ *S*(5) line is labelled on top of the spectrum.

The MIRI spectrum also reveals a wealth of water lines, particularly in the 7 μm spectral window (Fig. 3). This indicates the presence of a water reservoir in the terrestrial region of a disk already hosting two or more protoplanets. As such, it also provides important clues to theories on the origin of water during terrestrial planet formation in the solar system^23,24^. We focus on the ro-vibrational transitions of the bending mode of para- and ortho-water in the 7 μm region where the brightest lines are observed and contamination by the stellar atmosphere is negligible (Extended Data Fig. 3). This includes strong water blends dominated by lines with upper energy level  *E*_*u*_ ≃ 2,400–3,200 K. Weaker lines are also detected at the 1 mJy level, some of them corresponding to more excited levels up to *E*_*u*_ ≃ 4,300 K.

Further insight into the origin of water emission is obtained from zero-dimensional slab modelling, which has also been used to interpret Spitzer spectroscopic data^25^. The synthetic spectrum of water is calculated from a plane-parallel slab model, where the level populations are in local thermodynamic equilibrium (LTE) at a single excitation temperature *T*. The other fitting parameters are the line-of-sight column density *N* within an effective emitting area π*R*^2^ given by its radius *R*, and the intrinsic line broadening assumed to be *σ* = 2 km s^−1^ (ref. ^25^). Note that *R* does not need to correspond to a disk radius, but could also represent an annulus with the same area or an emission spot breaking the axisymmetry. The best-fit model is then obtained by minimizing the reduced *χ*^2^ between measured and model line fluxes over the individual spectral window around each H_2_O line (Extended Data Fig. 4).

The observed H_2_O spectrum in the 6.78–7.36 μm spectral region is best fitted with a slab of gas at *T* = 600 K, with an emitting area of radius *R* = 0.047 au and a column density of *N* = 1.4 × 10^18^ cm^−2^. The temperature is mostly determined by the ratio between the lines of different *E*_*u*_, for example, the series of lines in the 7.3 μm region. The column density is set by the ratio between the weaker lines and indicates that the brightest lines are optically thick. The emitting area is constrained by matching the fluxes of the optically thick lines, and points toward a compact emission region. This is further supported by the fact that the detected lines are broad (Δ*λ* ≈ 0.01–0.05 μm); if the line broadening is caused by the gas kinematics, the full-width half-maximum (FWHM) of the line would be about 100 km s^−1^, corresponding to a Keplerian radius of 0.1 au, consistent with the emitting area deduced from our fit after correction for disk inclination *i* = 51.7 ± 0.1^°^ (ref. ^11^). Interestingly, we find that our best-fit LTE model of the water emission in the 7 μm region reproduces water rotational lines at 15 μm reasonably well, suggesting that all water emission in the MIRI spectral range originates from inside approximately 0.05 au under LTE conditions (Extended Data Fig. 5).

Besides water in the 7, 15 and 17 μm regions, other species have been identified but the analysis is postponed to a future study. The fundamental Q-branch of CO_2_ corresponding to the *ν*_5_ bending mode is detected at 14.96 μm (Extended Data Fig. 5). Interestingly, the width of this feature is sensitive to the temperature and indicates cooler gas at *T* ≃ 200 K in the optically thin regime. The pure rotational molecular hydrogen H_2 _*S*(5) and H_2 _*S*(1) lines are detected at 6.91  and 17 μm (Fig. 1). We note that the H_2 _*S*(2), *S*(3) and *S*(4) lines coincide with the broad silicate emission feature and thus establishing their presence needs to await an in-depth analysis of this dust feature.

**Fig. 4:**
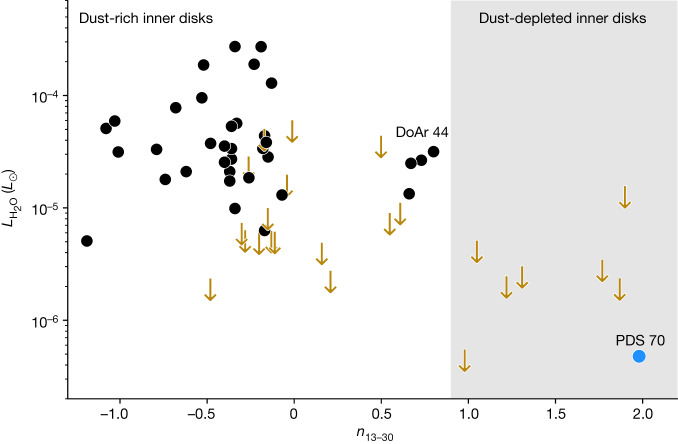
Comparison between water luminosity and mid-infrared spectral index (*n*_13–30_) for a sample^7^ of protoplanetary disks. *n*_13–30_ is a diagnostic of the presence and size of inner disk dust cavities: 0.9 < *n*_13–30_ < 2.2 corresponds to disks with large gaps and/or cavities^32,33^. Black dots represent disks with mid-infrared water detections. Disks for which only upper limits were obtained are shown as gold arrows. The grey shaded area highlights the location of dust-depleted inner disks with PDS 70 shown as a blue dot. The water line flux used to compute the water luminosity of PDS 70 is calculated as described in a previous work^7^. The Spitzer spectrum is used to estimate *n*_13–30_ for PDS 70 to be consistent with the other targets. Spitzer-IRS obtained only water luminosity upper limits for disks characterized by *n*_13–30_ greater than 0.9. Below 10 μm, IRS provided only a spectral resolution of *R*  ≈ 100, preventing a comprehensive view of water in the innermost regions^34^. DoAr 44 is a system schematically similar to PDS 70 (ref. ^29^). The two stars have comparable age and spectral types K3 and K7, respectively; DoAr 44 has a higher mass accretion rate of ${\dot{M}}_{{\rm{acc}}}\approx 1{0}^{-8}\,{M}_{\odot }$ yr^−1^ (ref. ^30^). The cavity size of DoAr 44 is 34 au (refs. ^7,29^), which is smaller than that of PDS 70 (approximately 54 au (ref. ^11^)). Both systems have small inner disks based on VLTI-GRAVITY^35^, VLT-SPHERE and ALMA data^8,11^, and in both systems the water emission is contained to within 1 au (ref. ^29^). The water luminosity of PDS 70 is two orders of magnitude weaker than that of DoAr 44, pointing to a colder water reservoir in PDS 70. This is consistent with the lower luminosity and lower accretion rate of PDS 70.

Spitzer-IRS observations detected water in approximately 50% of dust-rich inner disks around T Tauri stars^26^, but obtained only upper limits for disks with large inner dust gaps or cavities defined by a mid-infrared spectral index *n*_13–30_ > 0.9, where *n* is the slope of the spectrum between 13 and 30 μm  (Fig. 4)^7^. The detection of water vapour in the PDS 70 MIRI spectrum demonstrates that PDS 70 has maintained to some degree the physical and chemical conditions of dust-rich inner disks in its terrestrial planet-forming zone despite the presence of a notably large gap (see ‘Origin of water in PDS 70’ in Methods). Our LTE slab model only provides a first quantitative analysis of the H_2_O emission. Non-LTE effects could lead to subthermal line emission, which would make our estimated emitting area a lower limit. In T Tauri disks with strong radial temperature gradients, the water lines are expected to originate from different regions of the disk depending on their upper energy level and Einstein-*A* coefficients^27^. Detailed modelling using a realistic disk structure and including non-LTE effects such as infrared radiative pumping is needed in the future to further constrain the distribution of H_2_O across the inner disk. However, this first analysis already proofs that the inner disk of PDS 70 is rich in water and the inferred slab model parameters are roughly consistent with a detailed thermo-chemical model (ref. ^28^ and B. Portilla-Revelo, personal communication).

The luminosity of the 17 μm water lines is two orders of magnitude weaker for PDS 70 than DoAr 44 (refs. ^7,29^). DoAr 44 is a system with similar properties to PDS 70, but characterized by *n*_13–30_ < 0.9 (Fig. 4). This result points to a colder water reservoir in PDS 70, and is consistent with the lower luminosity and lower accretion rate of PDS 70 (refs. ^21,30^). This work opens a new window on the origin of water in protoplanetary disks by showing that MIRI MRS can now detect very weak (≲5 mJy) water lines in the innermost regions of disks with large gaps, and hence that the presence of water in the terrestrial planet-forming zone of dust-depleted inner disks is not as rare as previously thought.

## Methods

### PDS 70 system

PDS 70 (V1032 Cen) is a K7-type star in the Upper Centaurus-Lupus subgroup (*d* = 113.4 ± 0.5 pc (ref. ^36^)) in a late stage of accretion^22^ with an estimated age of 5.4 ± 1.0 Myr (ref. ^37^). The disk around PDS 70 (refs. ^38–40^) hosts two actively accreting protoplanets: PDS 70 b and PDS 70 c, which reside in an approximately 54 au annular gap between an inner and outer disk^8,9^. The presence of an inner dusty disk in the PDS 70 system has been inferred from both near-infrared scattered light and ALMA images^10–12^. The 855 μm dust continuum emission from the innermost disk regions is confined within the orbit of PDS 70 b (approximately 22 au; Extended Data Fig. 1), putting an upper limit to the inner disk radial extent of approximately 18 au (ref. ^12^). A population of small dust grains may be responsible for the observed inner disk emission although the current low dust mass estimates could support the simultaneous presence of small and large dust grains^41^.

### Observations and data reduction

The PDS 70 disk (CD-40-8434) was observed with MIRI^13,14^ on 1 August 2022 as part of the Guaranteed Time Observation (GTO) programme 1282 (PI: Th. Henning) with number 66. The disk was observed in FASTR1 readout mode with a four-point dither pattern in the negative direction for a total on-source exposure time of 4,132 s. The MRS^15^ mode was used, which has four IFUs. Each IFU (referred to as channel) covers a different wavelength range and splits the field of view into spatial slices. Calibration and processing of IFU observations produces three-dimensional spectral cubes. The latter are used to extract a final spectrum covering the MIRI 4.9–22.5 μm range and is a composite of the four IFUs: channel 1 (4.9–7.65 μm; *R* ≈ 3,400), channel 2 (7.51–11.71 μm; *R* ≈ 3,000), channel 3 (11.55–18.02 μm; *R* ≈ 2,400) and channel 4 (17.71–22.5 μm; *R* ≈ 1,600). Each channel is in turn composed of three sub-bands: SHORT (A), MEDIUM (B) and LONG (C) leading to a total of 12 wavelength bands.

We processed the PDS 70 data using a hybrid data reduction pipeline made from the combination of the JWST Science Calibration pipeline^42^ (v.1.8.4) stages 1 to 3, with dedicated routines based on the Vortex Image Processing (VIP) package^43,44^ for bad pixel correction, background subtraction and removal of spikes affecting the final spectrum. Specifically, data reduction proceeded as follows: (1) the class Detector1 of the JWST pipeline was used to process uncalibrated raw data files using Calibration Reference Data System (CRDS) context jwst_1019.pmap and default parameters; (2) apart from pixels flagged in the Data Quality (DQ) extension, we identified additional bad pixels with both an iterative sigma clipping algorithm and through a cross-shaped match filter, and corrected them using a two-dimensional Gaussian kernel; (3) Spec2 was then used with default parameters, but the background subtraction was skipped, and dedicated reference files^45^ for photometric and fringe flat calibrations were adopted; (4) as no dedicated background observation was taken, we leveraged the four-point dither pattern to obtain a first guess on the background map, then refined it using a median filter, which both smoothed the background estimate and removed residual star signals from it; (5) Spec3 was then run with default parameters, apart from the master_background and outlier_detection steps that were turned off, in the latter case to avoid spurious spectral features resulting from under-sampling of the point spread function; (6) we recentred the spectral cubes by applying the shifts maximizing the cross-correlation between cube frames, and found the location of the point spread function centroid with a two-dimensional Gaussian fit on the median image of each aligned cube; (7) spectra were then extracted with aperture photometry in 2.5-FWHM apertures centred on the centroid location (with the FWHM equal to approximately 1.22*λ*/*D* with the telescope diameter *D* equal to 6.5 m), corrected for both aperture size using correction factors^18^, and spikes affecting individual spaxels included in the aperture; (8) spectra were finally corrected for residual fringes at the spectrum level and the bands were stitched together based on the level of the shorter wavelength bands (these rescaling factors were systematically within 3% of the photometric solution). Spurious data reduction artefacts were masked at 5.12, 5.90, 7.45 and 7.50 μm. The uncertainty associated with each photometric measurement considers both Poisson and background noise, combined in quadrature. The former is an output from the JWST pipeline, whereas for the latter we propagated our background estimate obtained in step (4) through Spec3, and considered the standard deviation of the fluxes inferred in independent 2.5-FWHM apertures as a proxy for the background noise uncertainty. The final relative uncertainties range from approximately 0.1% to approximately 1.1% with respect to the continuum at the shortest and longest wavelengths considered in this work (4.9 and 22.5 μm, respectively).

### Local continuum fit

**Extended Data Fig. 2:**
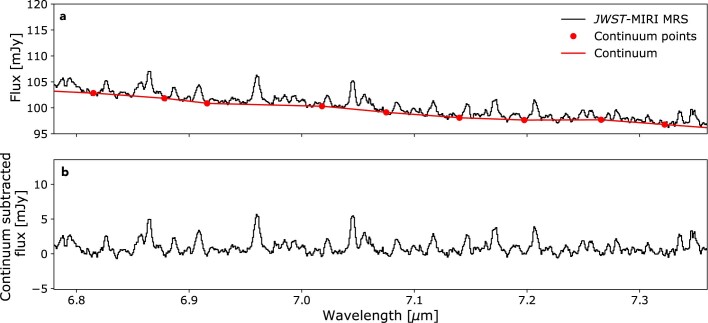
Local continuum fit used in the spectrum presented in Fig. 3. **a**, The selected continuum points are displayed as red dots and the interpolated continuum is shown as a red line. **b**, The continuum-subtracted spectrum.

Extended Data Fig. 2 shows the local baseline fit for the 7 μm region. The continuum level is determined by selecting line-free regions and adopting a cubic spline interpolation (scipy.interpolate.interp1d). This continuum is then subtracted from the original data to produce the spectrum shown in Fig. 3.

### Correction for the photospheric emission

**Extended Data Fig. 3:**
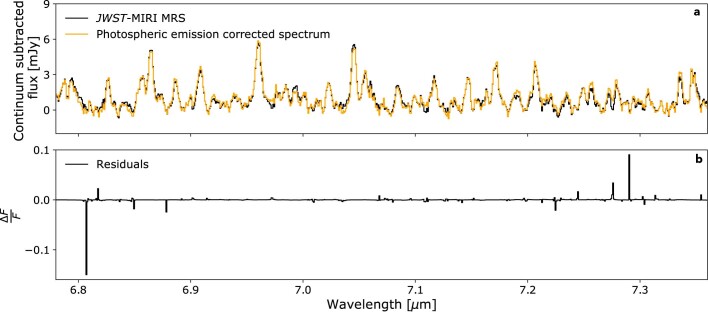
Correction for the photospheric emission. **a**, Comparison between the MIRI-MRS spectrum (black) and the spectrum corrected for the stellar photosphere (orange). Both spectra are continuum subtracted. **b**, The residuals show that the contamination from the stellar photosphere is negligible in the observed spectrum.

The observed near-infrared colour index, *J* − *K*_*s*_ = 1.01 (2MASS), indicates a small colour excess, *E*(*J* − *K*_*s*_) ≈ 0.16, which could be due to either interstellar extinction or a true excess in the *K*_*s*_ band, or a combination of the two. By assuming that the brightness in the *K*_*s*_ band is essentially due to photospheric emission we can, by using a model atmosphere provided by P. Hausschildt (personal communication, 2023), extrapolate the contribution into the mid-infrared spectral region. The parameters used for the model atmosphere of the PDS 70 K7-star are an effective temperature *T*_eff_ = 4,000 K, surface gravity log(*g*) = 4.5 and solar metallicity. At 5 μm the photospheric contribution amounts to 56 mJy, that is, 44% of the observed flux density. At longer wavelengths, in the 7 μm region in which the water emission is detected, the photosphere amounts to one-third of the observed flux density and the subtraction of the photospheric contribution marginally alters the continuum-subtracted spectrum (Extended Data Fig. 3).

### Slab models fits

The molecular lines are analysed using a slab approach that takes into account optical depth effects. The level populations are assumed to be in LTE and the line profile function to be Gaussian with a FWHM of Δ*V* = 4.7 km s^−1^ (*σ* = 2 km s^−1^)^25^. The line emission is assumed to originate from a slab of gas with a temperature *T* and a line-of-sight column density of *N*. Under these assumptions and neglecting mutual line opacity overlap, the frequency-integrated intensity of a line is computed as follows:

1 $$I=\frac{\Delta V}{2\sqrt{{\rm{l}}{\rm{n}}2}{\lambda }_{0}}{B}_{{\nu }_{0}}(T){\int }_{-{\rm{\infty }}}^{+{\rm{\infty }}}(1-\exp (-{\tau }_{0}{{\rm{e}}}^{-{y}^{2}})){\rm{d}}y,$$

where ${B}_{{\nu }_{0}}(T)$ is the Planck function, *λ*_0_ is the rest wavelength of the line and *τ*_0_ is the optical depth at the line centre *ν*_0_, with:

2 $${\tau }_{0}=\sqrt{\frac{{\rm{ln}}2}{{\rm{\pi }}}}\frac{{A}_{{\rm{ul}}}N{\lambda }_{0}^{3}}{4{\rm{\pi }}\Delta V}\left({x}_{{\rm{l}}}\frac{{g}_{{\rm{u}}}}{{g}_{{\rm{l}}}}-{x}_{{\rm{u}}}\right).$$

In this equation, *x*_l_ and *x*_u_ denote the level population of the lower and upper states, *g*_l_ and *g*_u_ their respective statistical weights and *A*_ul_ the spontaneous downward rate of the transition. The line intensity is then converted into integrated flux ${F}_{{\nu }_{0}}$ assuming an effective emitting area of π*R*^2^ and a distance to the source *d* as:

3 $${F}_{{\nu }_{0}}={\rm{\pi }}{\left(\frac{R}{d}\right)}^{2}I.$$

We note that neglecting mutual line overlap for H_2_O when calculating the line intensity is a valid approximation for N(H_2_O) ≲ 10^20^ cm^−2^ and significantly reduces the computational time^46^. Finally, the spectrum is convolved and sampled in the same way as the observed spectrum^47^ and all lines are then summed to prepare a total synthetic spectrum. The molecular data, that is, line positions, Einstein *A* coefficients and statistical weights stem from a previous work^48^.

### Fitting procedure for H_2_O vapour lines

**Extended Data Fig. 4:**
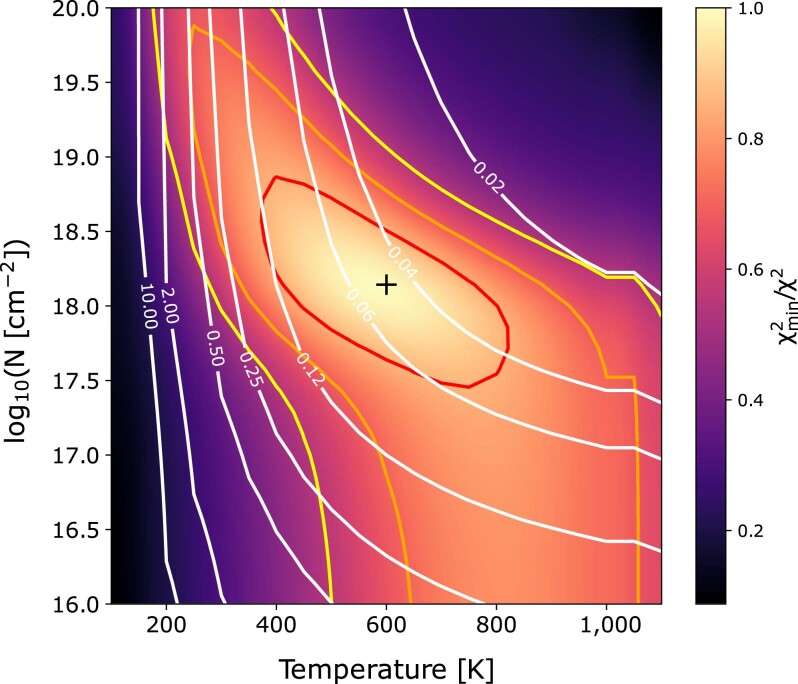
*χ*^2^ map for the fit of the 7 *μ*m region of the H_2_O bending mode. The best-fit model is represented by a black plus. The 1*σ*, 2*σ*, and 3*σ* confidence intervals are shown in red, orange, and yellow, respectively, for a typical noise level of *σ* = 0.15 mJy. The best-fitting emitting radius *R* for all values of *N* and *T* is indicated as white lines. In general, we find a degeneracy between a high *T* and low *N* solution, and a low *T* and high *N* solution. Within the framework of our LTE slab model, the data indicate mildly optically thick H_2_O emission at a temperature of about 600 K.

**Extended Data Fig. 5:**
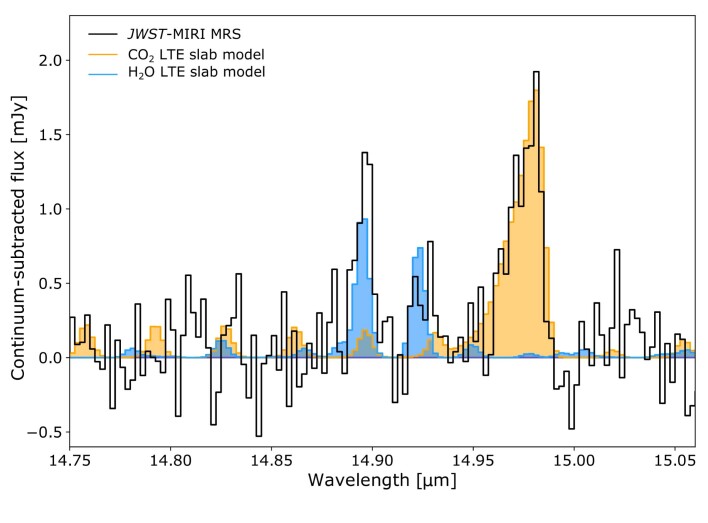
Continuum-subtracted spectrum in the 15 *μ*m region showing the detected Q-branch of CO_2_ (orange). The shape of this feature is sensitive to temperature and is well-fitted by an LTE slab model with *T*  ≃ 200 K. The strength of this feature can be reproduced with *N*(CO_2_) = 1.5 × 10^17^ cm^−2^ and *R* = 0.1 au. However, the aforementioned parameters are degenerate and are used for illustrative purposes only. Rotational lines of H_2_O (*J* = 14_5 10_ − 13_2 11_, *J* = 14_6 9_ − 13_3 10_; *E*_*u*_ ~ 4300 K) are also detected and they are reasonably well reproduced by the best-fit model for the 7 *μ*m region (blue). This could indicate that there is no additional reservoir of water at cooler temperature.

The LTE slab model described above is then used to fit the H_2_O lines in the 6.78–7.36 μm region following a *χ*^2^ method. First, an extended grid of models is computed varying the total column density from 10^15^ to 10^20^ cm^−2^ in steps of 0.17 in log_10_ space and the temperature from 100–1,400 K in steps of 50 K. We further assume an ortho-to-para ratio of 3. For each set of free parameters (*N*, *T*, *R*), a synthetic spectrum is calculated at the spectral resolving power *R *= 2,000 and rebinned to the spectral sampling of the observed spectrum using the slabspec python code^49^. The adopted spectral resolution is lower that the nominal MIRI MRS spectral resolution in channel 1 (ref. ^15^); it was selected to account for the observed line broadening. This spectrum is further used to compute the *χ*^2^ value on a spectral channel basis. Specific spectral windows are chosen to avoid contamination by other gas features. This includes all spectral channels falling within 0.02 μm (1,000 km s^−1^) of any hydrogen recombination line and a 0.01 μm wide spectral window at the position of the* S*(5) line of H_2_ at 6.91 μm. To mitigate the errors induced by the continuum subtraction procedure, we also include only spectral elements falling within 0.004 μm of a water line. For each value of (*N*, *T*), the *χ*^2^ is then minimized by varying the emitting size π*R*^2^. The resulting *χ*^2^ map is shown in Extended Data Fig. 4 together with the best-fit emitting radius. The confidence intervals are estimated following a previous work^50^ and using a representative noise level of *σ* = 0.15 mJy. We note that, owing to the large number of lines in this crowded region, there is little space to determine the noise on the continuum. Therefore, we estimate the noise level between 7.72 μm and 7.73 μm to avoid contamination by H_2_O and hydrogen  lines.

### Origin of water in PDS 70

At the typical densities of inner disk regions (*n*_H _≥ 10^8^ cm^−3^) the chemistry can rapidly reach steady-state conditions and water vapour can form from a simple reaction sequence involving O, H_2_ and OH. Water and OH absorb efficiently in the ultraviolet (that is, water and OH shielding), ensuring the survival of water molecules even in regions of reduced dust opacity^5^. This mechanism by itself is able to account for the column densities of water vapour detected in this work and is supported by the presence of CO_2_ emission. Small grains in the inner disk provide additional ultraviolet shielding. One question that naturally arises is whether the water vapour in PDS 70 originated before the formation of the giant protoplanets within the gap or whether there is a continuous supply of gas from the outer to the inner disk regions. ALMA high spatial resolution CO observations reveal the presence of gas inside the gap^11,51^. Observations and models find the gap to be gas depleted^11^ (two to three orders of magnitude assuming an *r*^−1^ surface density profile) and dust depleted^52^, but not empty. One possibility could be that a population of water-containing dust particles is able to filter through the orbits of PDS 70 b and PDS 70 c, enriching the inner disk reservoir^12^. Experimental evidence indicates that water chemically bound to complex silicates can be preserved to temperatures up to 400–500 K (refs. ^53,54^) and thus survive in the regions probed by our observations inside the water snowline. We note that some degree of dust filtering is expected with gas replenishment, as small dust particles can couple to the gas. Therefore, a replenishment of both gas and dust from the outer disk to sustain the water reservoir and hence the PDS 70 accretion rate is possible.

### Fitting procedure for the dust continuum

The 4.9–22.5 μm dust continuum is analysed using a two-layer disk model for the dust emission^55^. This model was successfully applied to Spitzer-IRS spectra of planet-forming disks^56^; we follow the same modelling approach here. We rebin the spectrum by averaging 15 spectral points and assign errors *σ* to the rebinned spectral points assuming a normal error distribution with equal weights for each individual spectral element. The stellar photospheric emission is represented by a stellar atmosphere model fitted to optical and near-infrared photometry. The disk model has three spectral components: (1) a hot inner disk *F*_rim_, (2) an optically thick midplane disk layer *F*_mp_ and (3) an optically thin warm disk surface layer *F*_sur_. The dust grains representing the disk components are assumed to have power-law temperature distributions, and each is characterized by a minimum and a maximum temperature *T*_atm_. The disk surface layer is assumed to consist of a number of dust species *i* with different chemical compositions and with a fixed number of grain sizes *j*, all emitting at the same temperatures. The total disk flux can then be written as:

4 $${F}_{\nu }={F}_{\nu ,{\rm{rim}}}+{F}_{\nu ,{\rm{mp}}}+{F}_{\nu ,{\rm{sur}}}$$

where

5 $${F}_{\nu ,{\rm{sur}}}=\mathop{\sum }\limits_{i=1}^{n}\mathop{\sum }\limits_{j=1}^{m}{D}_{\mathrm{i,j}}\,{\kappa }_{\mathrm{i,j}}{\int }_{{T}_{{\rm{atm,max}}}}^{{T}_{{\rm{atm,min}}}}\frac{2{\rm{\pi }}}{{d}^{2}}\,{B}_{\nu }(T)\,{T}^{\frac{2-{q}_{{\rm{atm}}}}{{q}_{{\rm{atm}}}}}{\rm{d}}T$$

and *B*_*ν*_(*T*) is the Planck function, *q*_atm_ is the power-law exponent for the temperature gradient in the disk surface layer, *κ*_*i*,*j*_ are the opacities in cm^2^ g^−1^ of dust species *i* with grain size *j*, *d* is the distance to the star and *D*_*i*,*j*_ are normalization factors^55^. We use three grain compositions (with SiO_2_, SiO_3_ and SiO_4_ stochiometry)^57–61^ and both amorphous and crystalline lattice structures to capture the rich spectral structure evident in the MIRI data. The choice of this set of compositions is based on previous analyses^56^, which showed that this set of materials is able to capture most spectral variations in planet-forming disks observed with Spitzer-IRS. We use either two or three grain sizes (that is, 0.1, 2 and 5 μm) for each of the dust species. In total, the model has 23 fitting parameters. We use the MultiNest Bayesian fitting algorithm^62^ and the PyMultiNest package^63^ to find the best-fit parameters. The resulting fit and the separate spectral components (star, inner rim, midplane and surface layer) are shown in Fig. 2.

### WISE time-series observations

**Extended Data Fig. 6:**
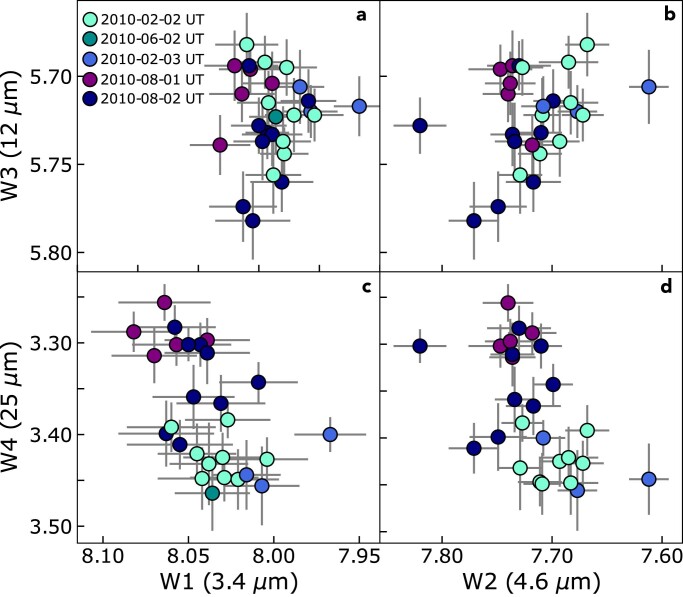
WISE Time-series photometry of PDS 70. Errorbars represent 1 s.d. **a**–**b**, WISE 3 (W3) is not anticorrelated with WISE 1 and WISE 2 due to the dominant 10 *μ*m silicate emission which does not vary substantially throughout different epochs. **c−d**, Anticorrelations are observed for WISE 1, WISE 2 and WISE 4 indicating a ’seesaw’-like time variability (see Methods for further details).

Extended Data Fig. 6 reports WISE time-series observations of PDS 70. Observations were executed on 2–3 February and 6 February 2010, and on 1–2 August 2010. We note that the source is highly variable and that WISE 4 (W4; 25 μm) is anticorrelated with WISE 1 (W1; 3.4 μm) and WISE 2 (W2; 4.6 μm). Such variability may be ‘seesaw’-like^19^, for which changes in the scale height of the inner disk wall shadow the disk material located further out. However, a complete ‘seesaw’ profile is not observed, as at wavelengths shorter than 8 μm the MIRI spectrum lies above the IRS spectrum (Fig. 1). This is not surprising as the wavelength of the ‘pivot’ point (that is, the wavelength at which a shift in emission is observed) is dependent on the location of the occulting material with respect to the star, the stellar luminosity and the inclination of the system, with highly inclined systems showing a more complete ‘seesaw’ than more face-on systems such as PDS 70 (*i* = 51.7 ± 0. 1^°^ (ref. ^11^)). Interestingly, WISE 3 (W3; 12 μm) is not anticorrelated with WISE 1 and WISE 2 because of the dominant 10 μm silicate band, which indeed shows a minor offset compared with the longer wavelengths. This indicates that the material contributing to the 10 μm emission is not shadowed. This behaviour is seen if the emission arises from warmer dust closer to the star than the occulting material or further above the disk midplane.

We also note that the difference in aperture size of Spitzer-IRS and MIRI MRS cannot explain the observed variability. The Spitzer-IRS low-resolution spectrograph has a slit width of 3.6*″* for wavelengths shorter than 14 μm and 10.2*″* for wavelengths longer than 14 μm. Although the maximum aperture of Spitzer-IRS at longer wavelengths is larger than that of MIRI MRS, this is not the case for the shorter wavelengths, for which the slit widths are similar for both observatories (approximately 3.6*″* versus 4.0*″*). However, a flux offset is also observed in this spectral region. In addition, in the case when the long wavelength excess would arise from an extended component, a jump in flux level at 14 μm—where the aperture size changes—would be present in the IRS data, but it is absent.

## Online content

Any methods, additional references, Nature Portfolio reporting summaries, source data, extended data, supplementary information, acknowledgements, peer review information; details of author contributions and competing interests; and statements of data and code availability are available at 10.1038/s41586-023-06317-9.

## Data Availability

The original data analysed in this work are part of the GTO programme 1282 (PI: Th. Henning) with number 66 and will become public on 2 August 2023 on the MAST database (https://mast.stsci.edu). The portion of the spectrum presented in Fig. 3 is available on Zenodo at https://zenodo.org/record/7991022. The spectroscopic data for water can be downloaded from the HITRAN database (https://hitran.org). The Spitzer-IRS spectrum plotted in Fig. 1 is part of the Spitzer-IRS GTO programme 40679 (PI: G. Rieke). The spectrum was extracted and calibrated using private codes^56,64^ and is available on Zenodo at https://zenodo.org/record/7991022. The optical constants of the dust species considered in the fitting procedure for the dust continuum can be downloaded from the HJPDOC database (https://www2.mpia-hd.mpg.de/HJPDOC).

## Code availability

The slab model used in this work is a private code developed by B.T. and collaborators. It can be obtained from B.T. upon request. The synthetic spectra presented in this work can be reproduced using the slabspec code, which can be found at 10.5281/zenodo.4037306. The fitting procedure for the dust continuum uses the publicly available MultiNest Bayesian fitting algorithm (https://github.com/JohannesBuchner/MultiNest) and the PyMultiNest package (https://github.com/JohannesBuchner/PyMultiNest). Figures were made with Matplotlib v.3.5.1. under the Matplotlib license at https://matplotlib.org/.
